# Inhibitory Effect of Oat Bran Ethanol Extract on Survival and Gemcitabine Resistance of Pancreatic Cancer Cells

**DOI:** 10.3390/molecules24213829

**Published:** 2019-10-24

**Authors:** Myoungjae Kim, Jeong-Geon Mun, Hyun Jin Lee, So-Ri Son, Mi-Ja Lee, Ji-Ye Kee

**Affiliations:** 1 Department of Oriental Pharmacy, College of Pharmacy, Wonkwang-Oriental Medicines Research Institute, Wonkwang University, 460 Iksandae-ro, Iksan, Jeonbuk 54538, Korea; 2 National Institute of Crop Science, Rural Development Administration, Crop Foundation Research Division, 181 Hyeoksinro, Isomyeon, Wanjugun, Jeonbuk 55365, Korea; 3 Department of Biomedical Science and Technology, Graduate School, Kyung Hee University, Kyungheedae-ro, Dongdaemun-gu, Seoul 02447, Korea

**Keywords:** Oat bran, pancreatic cancer, gemcitabine, avenacoside A

## Abstract

Pancreatic cancer (PC) is one of the most aggressive malignancies in the world. Gemcitabine (Gem), a nucleoside pyrimidine analogue, is a first-line chemotherapeutic drug for PC, but the tumor response rate of Gem is very low and resistance to Gem has emerged as a major problem in the treatment of PC. Oat bran, used as animal and human food, has been found to be beneficial to health. In this study, effects of oat bran ethanol extract (OBE) on PC cells and Gem-resistant PC cells were investigated in vitro. OBE decreased cell survival and colony forming ability of PC cells, without any cytotoxicity on the normal pancreatic cells. Flow cytometry analysis and TUNEL assay showed that the OBE reduced G1/S phase transition and induced death in PC cells through AMPK activation and downregulation of JNK. Additionally, OBE could overcome Gem resistance through reduction in RRM1/2 expression and showed synergistic effect by combinatorial treatment with Gem on Gem-resistant PC cells. Additionally, LC-MS data showed that avenacoside A was a component of OBE. Thus, this study elucidated the anti-proliferative effect of OBE and synergistic effect of OBE with Gem on PC cells and Gem-resistant cells.

## 1. Introduction

Pancreatic cancer (PC) is the one of the most aggressive and therapeutically ineffective malignant cancers. Mortality due to PC is fourth among the various cancer types worldwide [1]. In 2018, 458,918 new cases and 432,242 deaths by PC were recorded worldwide [2]. One of the primary reasons for the high mortality is the difficulty in early diagnosis of the disease because pancreas is behind the stomach and the patient shows general symptoms such as digestive problems, abdominal pain, and weight loss before metastasis. More than 80% of PC patients show metastasis at the time of diagnosis [3]. Despite various therapeutic methods, the 5-year survival rate of PC is only 8% and PC is projected to be the second leading cause of cancer deaths by 2030 [1,4].

Gemcitabine (Gem) is approved as a first-line chemotherapeutic drug for PC treatment. However, the response rate of Gem in PC patients is only 12% and most patients are resistant to Gem within a few weeks [5]. Less than 25% of PC patients benefit from Gem treatment and the median overall survival is only 6 months [6]. In order to overcome these limitations of Gem, combinatorial regimens such as folinic acid, 5-fluorouracil, irinotecan and oxaliplatin (FOLFIRINOX) have been widely used to treat PC patients [7,8]. FOLFIRINOX presents higher response rates and its median overall survival is nearly 12 months. However, this regimen shows considerable adverse effects such as lower blood counts, fever, infection, diarrhea, and weight loss [7]. Therefore, development of new chemotherapeutic drugs that can overcome chemoresistance and have less adverse effects on patients is needed.

Oat (*Avena sativa* L.) is an important cereal crop of the family of Poaceae grown worldwide [9]. Oats have various advantages; they require less nutrients to grow than the wheat or the corn [10,11]. Additionally, as people become more aware of their health, more people are consuming oats in the form of oatmeal, granola bars, cookies, and beverages. Recent studies have revealed that oats possess beneficial health effects on aging, oxidant, cancer, liver injury, hypercholesterolemia, and gastrointestinal problems [10,12,13,14,15,16].

In this study, the effects of ethanol extract from the oat bran (OBE) on PC were investigated in vitro. To confirm the anti-cancer effect of OBE on PC cell viability, colony formation, cell cycle distribution, apoptosis, and proteins were evaluated. Moreover, the combination effects of Gem and OBE on PC cells with acquired resistance to Gem was investigated to test if combination therapy could overcome drug resistance developed during cancer treatment.

## 2. Results

### 2.1. OBE Selectively Decreases Growth and Colony Formation Ability of PC Cells

**Figure 1:**
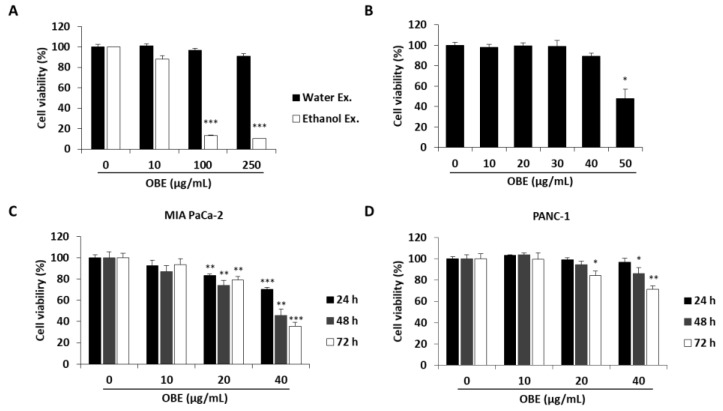

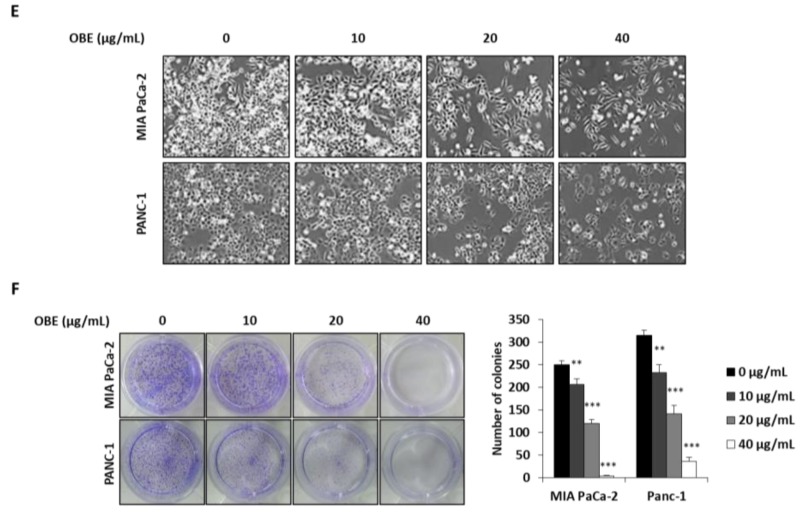
Effect of the ethanol extract of oat bran (OBE) on pancreatic cancer cells. (**A**) Viability of MIA PaCa-2 cells after treatment with water and ethanol extracts of oat bran. Cells (5 × 10^3^ cells/well) were seeded into a 96-well plate and treated with water and ethanol extracts of oat bran for 72 h. (**B**) HPNE cells (5 × 10^3^ cells/well) were seeded into a 96-well plate and treated with various concentrations of OBE for 72 h. (**C** and **D**) MIA PaCa-2 (**C**) and PANC-1 (**D**) cells (5 × 10^3^ cells/well) were seeded into a 96-well plate and treated with OBE (0–40 μg/mL) for 24–72 h. Cell viability was measured using WST reagent. (**E**) Morphology of OBE-treated MIA PaCa-2 and PANC-1 cells after 72 h. (**F**) Colony formation of OBE-treated MIA PaCa-2 and PANC-1 cells after 7 days. Data represent the mean of three experiments analyzed through Student’s t-test. * *p* < 0.05, ** *p* < 0.01, and *** *p* < 0.001.

To determine the anti-proliferative effect of oat bran water and ethanol extracts, various concentrations of both extracts were used to treat MIA PaCa-2 cells for 72 h. Oat bran ethanol extract (OBE) significantly decreased the survival of MIA PaCa-2 cells, while water extract of the oat bran did not change the cell viability (Figure 1A). hTERT-immortalized human pancreatic epithelial nestin-expressing (HPNE) cells, which are derived from normal pancreatic duct, were treated with OBE for 72 h to investigate the selective cytotoxicity of OBE on the PC cells. At less than 40 µg/mL, OBE showed no cytotoxicity on HPNE cells (Figure 1B). Various concentrations of OBE (0–40 µg/mL) were used to treat PC cell lines including MIA PaCa-2, and PANC-1 for 24–72 h. As seen in WST assay results, OBE inhibited cell viability of PC cells in a dose- and time-dependent manner (Figure 1C,D). Changes to the cell morphology were observed under a microscope after OBE treatment for 72 h (Figure 1E). Additionally, colony formation ability of MIA PaCa-2 and PANC-1 cells was reduced by OBE treatment (Figure 1F). Thus, OBE can selectively suppress growth and colony formation ability of PC cells.

### 2.2. OBE Inhibits Proliferation of PC Cells by Inducing G0/G1 Phase Arrest

**Figure 2:**
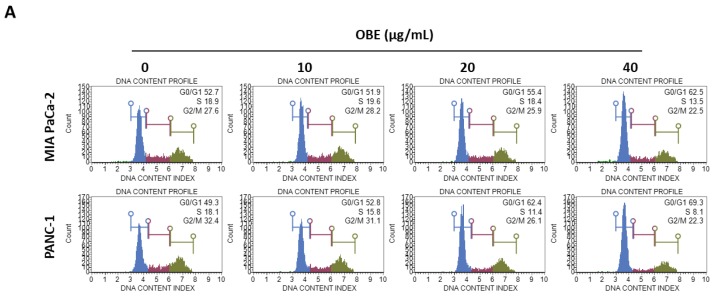

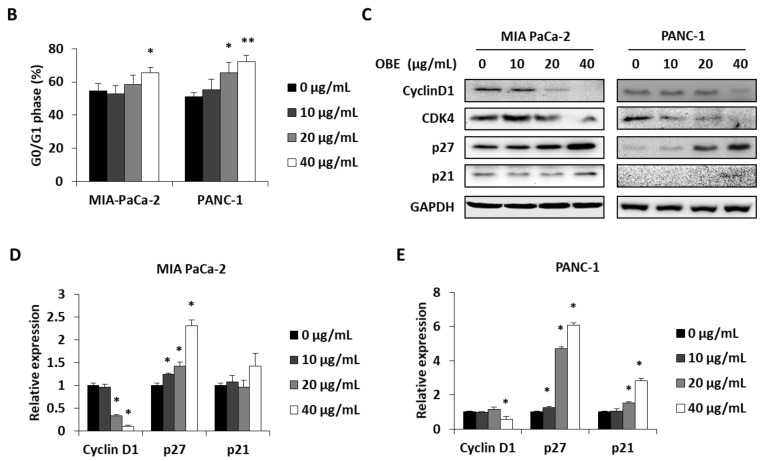
Effect of OBE on cell cycle arrest of MIA PaCa-2 and PANC-1 cells. MIA PaCa-2 and PANC-1 cells were seeded into 6-well plates and incubated for 72 h with OBE. (**A**) Analysis of cell cycle phase distribution. (**B** and **C**) Percentages of cell cycle phase distribution in MIA PaCa-2 (**B**) and PANC-1 (**C**) cells. (**D** and **E**) Protein levels of G0/G1 phase arrest-related factors in OBE-treated PC cells. Data represent the mean of three experiments analyzed through Student’s t-test. * *p* < 0.05, ** *p* < 0.01, and *** *p* < 0.001.

In general, cell proliferation is regulated by the progression of the cell cycle phase. Therefore, the effect of OBE on cell cycle distribution was analyzed. OBE interfered in the G1/S phase transition in MIA PaCa-2 and PANC-1 cells (Figure 2A). Percentages of G0/G1 phase cells increased from 51.75% to 62.05% in the MIA PaCa-2 cells and from 49.55% to 70.2% in the PANC-1 cells (Figure 2B). Cyclin D1 can activate the G1/S transition of the cell cycle phase, and cyclin-dependent kinase (CDK) inhibitors such as p21 and p27 are known to prevent inadequate cyclin/CDK activity at the G1 phase [17,18,19]. 

Protein levels of G1/S phase transition regulators were determined to elucidate the mechanism of OBE-induced G0/G1 phase arrest (Figure 2C). Expression of cyclin D1 and CDK4 was decreased by OBE treatment, whereas p21 and p27 expression was increased in both the cell lines (Figure 2D,E). These data showed that OBE can induce cell cycle arrest at the G0/G1 phase of MIA PaCa-2 and PANC-1 cells.

### 2.3. OBE Induces Apoptotic Cell Death of PC Cells

**Figure 3:**
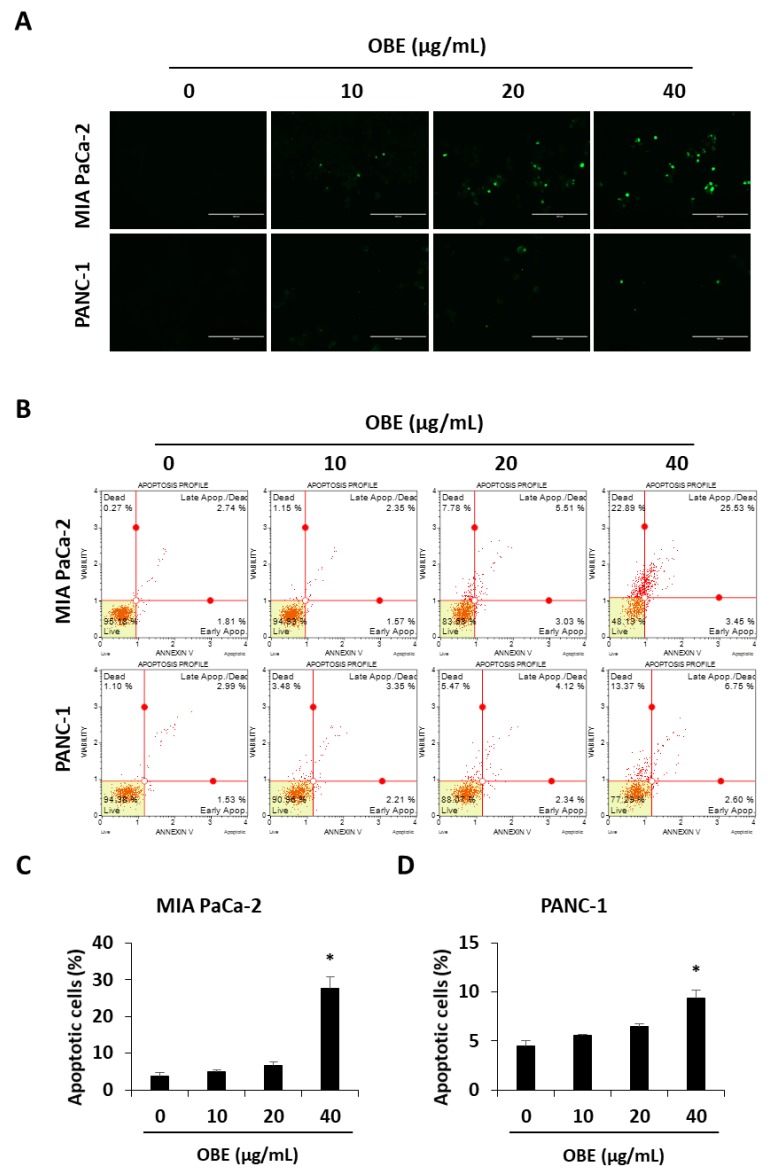
Effect of OBE on apoptosis in MIA PaCa-2 and PANC-1 cells. (**A**) TUNEL staining in OBE-treated PC cells. Cells were photographed at 200x magnification. (**B**) Apoptosis of OBE-treated PC cells was detected by annexin V assay. (**C** and **D**) The percentage of apoptotic cells in OBE-treated MIA PaCa-2 (**C**) and PANC-1 (**D**) cells. Data represent the mean of three experiments analyzed through Student’s t-test. * *p* < 0.05, ** *p* < 0.01, and *** *p* < 0.001.

The ability to avoid apoptosis and sustain survival is one of the basic characteristics of cancer cells [20]. Apoptosis is a tightly controlled programmed cell death that can be induced by chemotherapeutic drugs. In this study, it was investigated whether OBE-induced cell death was related to apoptosis in MIA PaCa-2 and PANC-1 cells. OBE treatment increased the TUNEL-positive cells in MIA PaCa-2 and PANC-1 cells in a dose-dependent manner (Figure 3A). AnnexinV/PI staining results showed that the OBE increased apoptosis in MIA PaCa-2 and PANC-1 cells (Figure 3B–D). 

**Figure 4:**
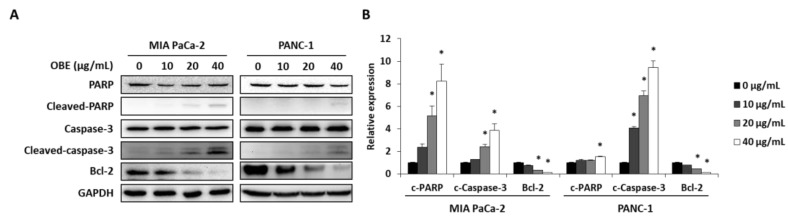

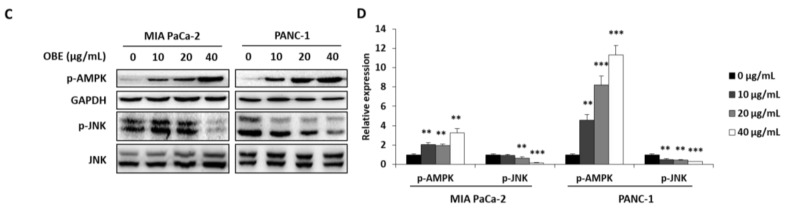
Effect of OBE on apoptosis-related proteins in MIA PaCa-2 and PANC-1 cells. MIA PaCa-2 and PANC-1 cells treated with OBE for 72 h were harvested and target proteins were detected by western blotting. (**A**) Apoptosis-related proteins in OBE-treated PC cells. (**B**) Density of apoptosis-related proteins band was analyzed by Image J. (**C**) Phosphorylation of AMPK and JNK in OBE-treated PC cells. (**D**) Density of AMPK and JNK phosphorylation band was determined by Image J. Data represent the mean of three experiments analyzed through Student’s t-test. * *p* < 0.05, ** *p* < 0.01, and *** *p* < 0.001.

In addition, the expression levels of apoptosis-related proteins were determined. OBE increased the cleavage of PARP and caspase-3, whereas Bcl-2 expression was decreased by OBE (Figure 4A,B). Since activation of AMPK and JNK is involved in the regulation of cancer cell proliferation and apoptosis [21,22], the phosphorylation of AMPK and JNK in OBE-treated PC cells was determined. OBE increased phosphorylation of AMPK, whereas JNK phosphorylation was reduced (Figure 4C,D). These results revealed that the OBE can reduce viability of PC cells by inducing AMPK and JNK-mediated apoptosis.

### 2.4. OBE Inhibits Viability of Gem-Resistant PC Cells by Reducing RRM1 and RRM2 Expression

**Figure 5:**
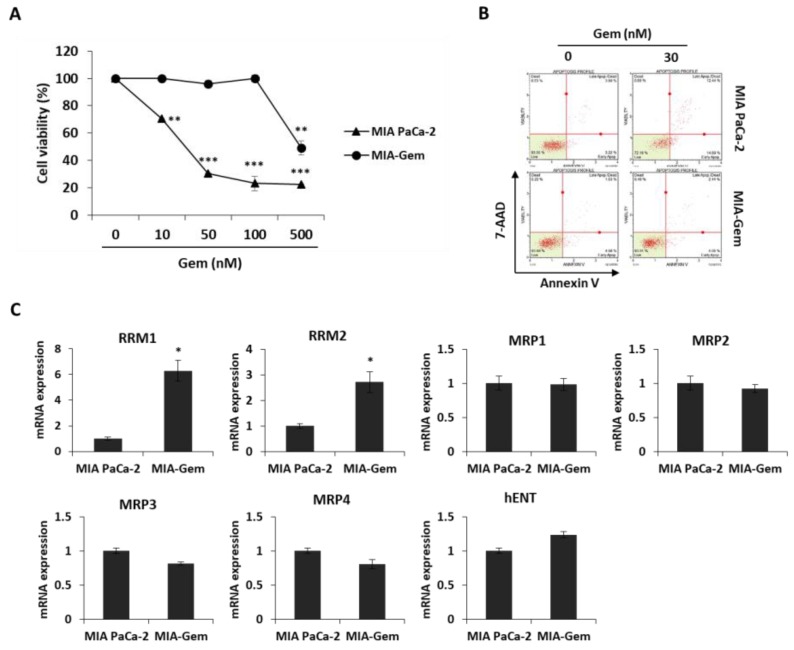

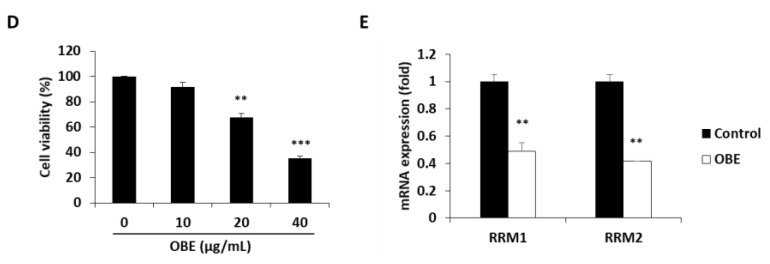
Effect of OBE on the Gem-resistant PC cells. (**A**–**C**) Establishment of MIA-Gem cell line that consists of Gem-resistant PC cells. (**A**) Viability of MIA PaCa-2 and MIA-Gem cells after Gem treatment for 72 h. (**B**) Apoptotic cells of MIA PaCa-2 and MIA-Gem cells after Gem (30 nM) treatment for 72 h. (**C**) Expression of drug resistance-related factors including RRM1, RRM2, MRPs, and hENT was measured by real-time RT-PCR. (**D**) Viability of OBE-treated MIA-Gem cells. (**E**) mRNA expression of RRM1 and RRM2 in OBE-treated MIA-Gem cells. Data represent the mean of three experiments analyzed through Student’s t-test. * *p* < 0.05, ** *p* < 0.01, and *** *p* < 0.001.

Most patients with PC are reported to be resistant to Gem within a few weeks of starting the chemotherapy regimen [5] and this resistance drastically reduces the cure rate of cancer patients. Therefore, overcoming resistance to anticancer agents is one of the efficient methods to treat PC patients. Gem-resistant PC cells were established to investigate the effect of OBE on Gem resistance. The IC_50_ values for Gem on the MIA PaCa-2 and the Gem-resistant MIA PaCa-2 (MIA-Gem) cells were 29 ± 0.3 nM and 350 ± 3.3 nM, respectively (Figure 5A). In addition, at 30 nM of Gem treatment, apoptosis was seen in MIA PaCa-2, but not in MIA-Gem cells (Figure 5B). To find the molecules related to Gem-resistance in PC cells, expression of several drug resistance-related molecules was determined. Among these molecules, drug metabolizer regulators, RRM1 and RRM2 mRNA expression levels were significantly higher in the MIA-Gem cells than in the MIA PaCa-2 cells. 

However, expression of influx pump and efflux pump-related genes such as MRP1-4 and hENT did not change in the MIA-Gem cells compared to the MIA PaCa-2 cells (Figure 5C). These results indicated that RRM1 and RRM2 are involved in the Gem-resistance of PC cells.

To investigate effect of OBE on Gem-resistant PC cells, OBE used to treat the MIA-Gem cells. Notably, OBE significantly decreased the cell viability of MIA-Gem cells at non-toxic concentrations on normal pancreas cells (Figure 5D). RRM1 and RRM2 expressions in OBE-treated PC cells were measured to determine whether OBE can overcome Gem-resistance of the PC cells. mRNA levels of RRM1 and RRM2 were decreased in the OBE-treated MIA-Gem cells (Figure 5E). These data suggested that OBE might overcome Gem-resistance of PC cells through the regulation of RRM1 and RRM2 expression.

### 2.5. Combination of Gem and OBE Shows Synergistic Inhibitory Effect on Survival of Gem-Resistant PC Cells

**Figure 6:**
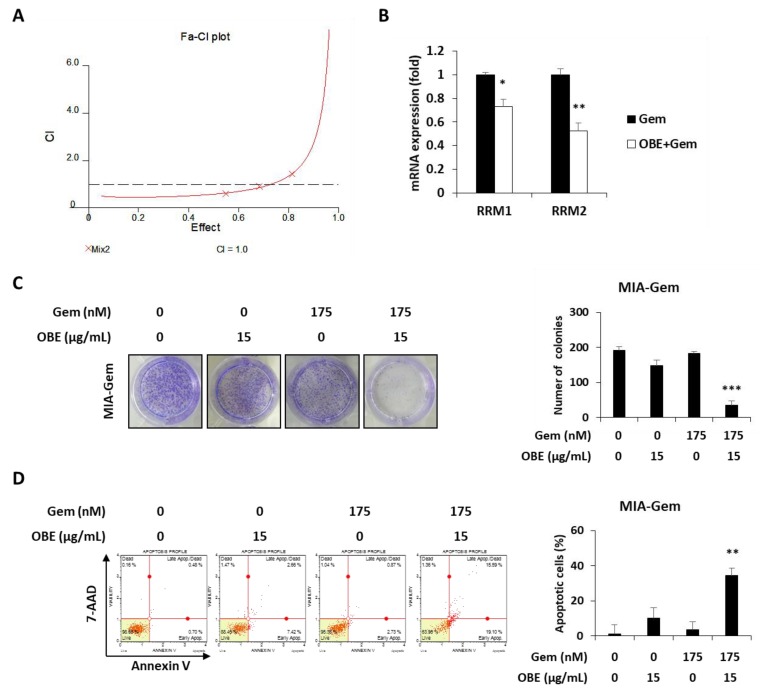
Effect of OBE and Gem combination on Gem-resistant PC cells. (**A**) OBE elicits synergistic effect with Gem. CI-effect plots were generated using CalcuSyn software. (**B**) Changes in RRM1/2 expression after OBE (15 µg/mL) and Gem (175 nM) combination treatment. (**C**) Colony formation. MIA-Gem cells were seeded into 12-well plates and stabilized. OBE, Gem, and their combination were used to treat the cells for 7 days. (**D**) Annexin V assay was conducted using OBE, Gem, and their combination-treated MIA-Gem cells. Data represent the mean of three experiments analyzed through Student’s t-test. * *p* < 0.05, ** *p* < 0.01, and *** *p* < 0.001.

Combination therapy, a treatment combining two or more drugs, has been commonly used to improve overall survival of PC patients [23]. It was investigated whether there is a synergistic effect of OBE in combination with Gem. Based on CI values, 15 μg/mL of OBE plus 175 nM of Gem combination showed better synergistic effect than the other concentrations of OBE and Gem together (Figure 6A and Table 1). It was hypothesized that this synergistic effect of OBE and Gem combination on Gem-resistant PC cells might be due to the regulation of RRM1 and RRM2 expressions. As expected, treatment with OBE and Gem combination significantly decreased expression of RRM1 and RRM2 in MIA-Gem cells (Figure 6B). Moreover, the synergistic effect of OBE with Gem combination was confirmed on MIA-Gem cells. Combinatorial treatment revealed higher inhibitory effect on colony formation and apoptosis in MIA-Gem cells than with OBE or Gem treatment alone (Figure 6C,D). These results demonstrated that the OBE and Gem combination has synergistic effect on the viability of Gem-resistant PC cells.

## 3. Discussion

This study investigated the effect of OBE on the survival and Gem resistance of PC cells using in vitro experiments. Restriction of cell cycle progression is one of the effective strategies for cancer treatment. Cell cycle consists of series of events leading to cell division and replication associated with cell proliferation. Cell cycle progression could be suppressed when DNA replication is not appropriate due to DNA damage and nutrient depletion [24]. Cyclin/CDKs and CDK inhibitors interact with each other to regulate cell cycle transitions. [25,26]. Cyclin D1 is a cell cycle activator that complexes with CDK2 and CDK4 to induce G1/S transition [17]. Among the CDK inhibitors, p21 inhibits the formation of the cyclin-CDK2 complex, and p27 directly inhibits CDK4, resulting in arrest at the G1 phase of the cell cycle [18,19]. In this study, the OBE induced G0/G1 phase arrest in both MIA PaCa-2 and PANC-1 cells by regulating expression of cyclin D1, CDK4, p21, and p27.

Apoptosis, or programmed cell death, plays a crucial role in the development and maintenance of homeostasis [27]. Various kinds of anti-cancer drugs inhibit cancer progression by directly inducing apoptosis in cancer cells [28]. In apoptotic pathway, caspase-3 plays a crucial role in the activation of apoptosis by cleaving various key cellular proteins including Bcl-2, which normally prevents apoptosis in cancer cells [29]. Cleavage of the Bcl-2 protein can promote further activation of caspases, which ultimately leads to apoptotic cell death [30,31]. PARP, which can repair damaged DNA, is also cleaved and loses its function when the caspases are degraded [31,32]. In the present study, OBE induced apoptosis in MIA PaCa-2 and PANC-1 cells through the induction caspase-3 and PARP cleavage and by reducing Bcl-2 expression.

AMPK is a serine/threonine protein kinase and it is a sensor of cellular energy and nutritional status. AMPK plays an essential role in the metabolic pathways and regulates cell survival and death through the modulation of energy homeostasis. Activation of AMPK is known to decrease tumor growth by inhibition of cancer cell proliferation [21]. JNK is involved in diverse biological functions including apoptotic mechanisms, cell cycle regulation, and cell survival. Notably, inhibition of JNK activity can inhibit tumor formation through promotion of apoptosis [22]. In this study, OBE increased AMPK activation and decreased JNK phosphorylation in both MIA PaCa-2 and PANC-1 cells. Although AMPK is known as autophagy inducer in various kinds of cancers, OBE did not increase autophagy-related factors such as LC3B and beclin-1 in this study (Data not shown). These results indicated that the OBE-induced apoptosis was mediated by AMPK and JNK phosphorylation.

Since drug resistance leads to low efficacy for chemotherapy in PC patients, several combination therapies to overcome the Gem-resistance have been attempted [23]. Gem is a deoxycytidine nucleoside analog, and its structure is similar to deoxyribonucleotides, which are essential for DNA synthesis and repair. Thus, Gem exhibits anti-cancer effect by interfering with DNA repair of cancer cells [33]. Among the drug resistance-related molecules, RRM1/2 are multimeric enzymes that convert ribonucleotides to deoxyribonucleotides and are involved in repairing damaged DNA [34,35]. RRM1/2 are predictive markers of Gem response in PC because high expression of RRM1/2 in PC patients is associated with lower susceptibility to Gem chemotherapy [36,37]. In MIA-Gem cells, RRM1 and RRM2 expression was significantly increased while other factors did not change. OBE could inhibit the viability of MIA-Gem cells by downregulation of RRM1 and RRM2. Moreover, the use of OBE and Gem together showed synergistic effects on Gem-resistant PC cells through inhibition of colony formation and promotion of apoptosis. This synergistic effect of combination is also presented by regulating RRM1 and RRM2 in MIA-Gem cells.

**Figure 7:**
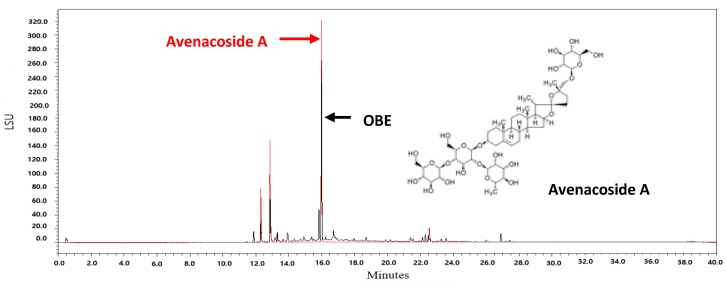
UPLC chromatograms of OBE and avenacoside A.

Oat bran contains 16 kinds of steroidal saponins [38]. Among these compounds, avenacoside A has been shown to inhibit the growth of HCT116 and HT29 human colon cancer cells and decrease the production of intracellular IL-2 in activated T cells [38,39]. Since avenacoside A was found in OBE by UPLC analysis (Figure 7), it is hypothesized that the anti-proliferative effect of OBE on PC cells might be due to avenacoside A. The content of avenacoside A in OBE was 1.075 mg/g. Further studies are needed that investigate the inhibitory effect of OBE at proper concentrations on survival and Gem-resistance in PC cells.

## 4. Materials and Methods

### 4.1. Antibodies and Reagents

EZ-cytox cell viability assay kit was purchased from DoGen (Seoul, Korea). Muse cell cycle kit, Muse annexin V, and dead cell assay kit were purchased from the Millipore (Bedford, MA, USA). Gemcitabine and crystal violet solution were purchased from Sigma-Aldrich (St. Louis, MO, USA). In situ cell death detection kit, fluorescein was purchased from Roche (Mannheim, Germany). Enhanced chemiluminescence regent was purchased from Santa Cruz Biotechnology, Inc. (Dallas, TX, USA). Trizol reagent and cDNA synthesis kit were purchased from Invitrogen Inc. (Carlsbad, CA, USA). Power SYBR^®^ Green PCR master mix was purchased from Applied Biosystems (Foster City, CA, USA). Antibodies against CDK4, cyclin D1, p21, p27, GAPDH, PARP, caspase-3, Bcl-2, phospho-AMPK, and AMPK were purchased from Cell Signaling (Danvers, MA, USA). Phospho-JNK and JNK antibodies were purchased from Santa Cruz.

### 4.2. Preparation of Oat Bran Extracts

Oat bran (*Avena Sativa* L.) was purchased from Jeongeup City Oats Company (Jeongeup-si, Jeonbuk, Korea) in 2017. Oat bran was ground in a laboratory test mill (Brabender Technologie, Duisburg, Germany). The flour (100 g) was defatted 3 times with hexane (1 L) for 24 h at room temperature. After filtration with filter paper (Whatman No. 3), the residual oat bran was extracted 3 times with prethanol (1 L) and filtrated by means of a Buechner funnel lined with filter paper (Carl Roth, Karlsruhe, Germany, 111A, Ø100 mm). The filtrates were combined and concentrated in a rotary evaporator. The residual from the prethanol extract was extracted further two times with 1 L water for 24 h and dried with a freeze dryer. At that time, the extraction yield was about 2%.

### 4.3. Cell Culture

Human pancreatic duct cell line, HPNE and human PC cell lines, MIA PaCa-2 and PANC-1 were purchased from the American Type Culture Collection (ATCC, Manassas, VA, USA). HPNE was cultured in the complete growth medium made according to the information provided with HPNE by ATCC. MIA PaCa-2 and PANC-1 cells were cultured in DMEM (Hyclone, Marlborough, MA, USA). All media were supplemented with 10% fetal bovine serum (FBS) and 1% penicillin/streptomycin (Invitrogen Inc.). Cells were maintained at 37 °C in humidified incubator containing 5% CO_2_.

### 4.4. Establishment of Gem-Resistant MIA PaCa-2 Cells

To establish MIA-Gem cells, the half maximal inhibitory concentration (IC_50_) of Gem at 72 h was determined using the WST-1 reagent. MIA PaCa-2 cells were exposed to IC_50_ of Gem for 72 h, and then grown until 80% full in fresh medium without Gem. During subculture, MIA PaCa-2 cells were exposed to gradually increasing concentrations of GEM from 30 to 500 nM for 8 months.

### 4.5. Cell Viability

HPNE, MIA PaCa-2, PANC-1, and MIA-Gem cells were seeded in 96-well plates at a density of 5 × 10^3^ cells/well. After stabilization, fresh media with various concentrations of OBE (0–50 µg/mL) was added to the cells. Then, cells were incubated for 24–72 h. Subsequently, 10 µl of water soluble tetrazolium salt (WST-1) reagent was added, and the plates were incubated for another 2 h. Absorbance was measured at 450 nm with a microplate reader. Cell viability was calculated from the mean value of three wells.

### 4.6. Colony Formation Assay

Cells (5 × 10^3^ cells/well) were seeded into 12-well plates. After overnight incubation, OBE was added into the wells and further incubated for another 7 days. The medium was refreshed every 3 days. For crystal violet staining of the colonies, cells were fixed with 4% paraformaldehyde for 15 min, and then washed twice with phosphate buffered saline (PBS). Colonies were stained by 0.1% of crystal violet solution for 30 min and rinsed twice with PBS.

### 4.7. Flow Cytometric Detection of Cell Cycle

Cell cycle analysis was carried out using the Muse Cell Cycle Kit according to the manufacturer’s instructions. Cells were seeded in 6-well plates at density of 3 × 10^5^ cells/well and incubated for 24 h then treated with OBE for 72 h. Following cell harvest, cells were fixed with 75% ethanol at −20 °C for at least overnight. Cells were washed with PBS and resuspended in 150 µL of Cell Cycle Reagent at room temperature in the dark for 30 min. After staining, cell cycle phase distribution was analyzed and quantified using Muse Cell Analyzer.

### 4.8. Annexin V Assay

Annexin V assay was conducted using Muse annexin V and dead cell assay kit according to the manufacturer’s instructions. Cells (5 × 10^4^ cells/well) were seeded into 6-well plates and treated with OBE for 72 h. Harvested cells (1 × 10^5^ cells/100 µL) were stained with 100 µL of Muse annexin V reagent at room temperature for 20 min in the dark. The samples were analyzed using Muse cell analyzer.

### 4.9. TUNEL Assay

Apoptotic cells were confirmed using in situ cell death detection kit, Fluorescein following to the manufacturer’s instructions. In brief, the cells were seeded into a 8-well chamber slide (5 × 10^3^ cells/well) for 24 h and treated with OBE for 72 h. Cells were fixed with 4% paraformaldehyde for 1 h and permeabilized using 0.1% Triton X-100 in 0.1% sodium citrate on ice for 2 min. TUNEL reaction mixture was added to chamber slide and incubated for 1 h in the dark. TUNEL-positive cells were washed with PBS and visualized by fluorescence microscope (Carl Zeiss, Oberkoche, Germany).

### 4.10. Western Blot Analysis

Total proteins from cells were extracted using RIPA buffer containing phosphatase inhibitor, and the protein concentrations were determined using a BCA protein assay. Protein samples with sample buffer were loaded into SDS-PAGE gels and separated by electrophoresis. After transfer to PVDF membrane, 5% skim milk was used to block for 1 h. The membrane was incubated overnight with primary antibodies and washed with 0.1% PBST. After incubation with horseradish peroxidase (HRP)-conjugated secondary antibodies for 1 h, an enhanced chemiluminescence reagent was used for detection. Photographs of the blots were acquired using a FluoroChem E image analyzer (Cell Bioscience, Santa Clara, CA, USA). The densitometric analysis was performed using ImageJ software (National Institutes of Health, Bethesda, MD, USA) to quantify the protein bands.

### 4.11. Real-Time RT-PCR

Total RNA was extracted using Trizol Reagent according to the manufacturer’s protocol. First-strand complementary DNA (cDNA) was synthesized using cDNA synthesis kit according to the manufacturer’s protocol. Real-time RT-PCR was performed using the Power SYBR^®^ Green PCR master mix and Step-One Plus^TM^ real-time PCR system (Applied Biosystems). The sequence of the primers as follows: RRM1, 5′-GGCACCCCGTATATGCTCTA-3′ and 5′-CCAGGGAAGCCAAATTA CAA-3′; and RRM2, 5′-ACAGAAGCCCGCTGTTTCTA-3′ and 5′-CCCAGTCTGCCTTCTTCTTG-3′; and GAPDH, 5′-CTGCACCACCAACTGCTTAG-3′ and 5′-TTCAGCTCAGGGATGACCTT-3′. Results were expressed as an average of the triplicate samples of three independent experiments.

### 4.12. Evaluating Drug Interactions

CalcuSyn software was used to evaluate drug interactions between OBE and Gem. It utilizes the combination index (CI) method derived from the median-effect principle established by Chou and Talalay [40]. CI equation is as follows:

(1) $$\mathrm{CI}\;=\;\frac{D_{A}{}_{+B}}{{{(D}_{50})}_{A}}\;+\;\frac{D_{A}{}_{+B}}{{{(D}_{50})}_{B}}\;+\;\alpha \frac{D_{A}{}_{+B}}{{{(D}_{50})}_{A}{{(D}_{50})}_{B}}$$

where D_A+B_ is the concentration for Drug A plus Drug B, resulting in 50% inhibition compared to the control. Where (D_50_)_A_ and (D_50_)_B_ are the concentrations for Drug A and Drug B separately, resulting in 50% inhibition compared to the control. When Drug A and B are mutually exclusive, α is 0 and when they are non-exclusive, α is 1. It was considered synergism when CI < 0.8, additive effect when 0.8 < CI < 1.2 and antagonism when CI > 1.2.

### 4.13. UPLC Analysis

For UPLC analysis, 1.3 μL aliquots were injected on an UPLC system (Acquity Ultra Performance LC, Waters, Milford, MA, USA) with ELS detector. The LC was controlled by MassLynx software (version 4.1). The column was a Halo C18 (2.1 × 100 mm, 2.7 μm). The mobile phase was A: 0.1% formic acid in DW and B: acetronitrile. The flow rate was 0.5 mL/min and gradients were as follows: 0–3 min, 97% A; 3–10 min, 97–85% A; 10–15 min, 85–70% A; 15–20 min, 70–50% A; 20–30 min, 50–10% A; 30–37 min, 10% A. The column and sampler temperature were 35 °C and 15 °C, respectively. Gas pressure of ELS detector was 40.0 psi.

### 4.14. Statistical Analysis

All experiments were conducted at least three times. The data represent the mean ± standard deviation of three independent experiments. Statistically significant differences between control and treatment groups were determined by Student’s t-test. SPSS Statistics v18 (IBM Crop., Armonk, NY, USA) was used as the statistical analysis software. A *p-*value of less than 0.05 was considered statistically significant.

## 5. Conclusions

The present study demonstrated that OBE selectively decreased the viability of PC cells by inducing cell cycle arrest and apoptosis via AMPK and JNK phosphorylation. OBE also inhibited survival of MIA-Gem cells through downregulation of RRM1 and RRM2. Combination treatment with OBE and Gem decreased colony formation and increased apoptosis in MIA-Gem cells. Therefore, OBE and Gem combination revealed synergistic effects on MIA-Gem cells. Taken together, OBE might be an effective agent to treat PC by overcoming drug resistance.

## Figures and Tables

**Table 1:** Combinatory index values (CI), symbols, and descriptions for classifying synergism or antagonism using the CalcuSyn software.

OBE (µg/mL)	Gem (nM)	CI	Symbol	Description
15	175	0.47	+++	Synergism
30	350	0.76	++	Moderate synergism
60	700	1.2	-	Slight antagonism

## References

[1] Siegel R.L., Miller K.D., Jemal A. (2018). Cancer statistics 2018. CA Cancer J. Clin..

[2] Bray F., Ferlay J., Soerjomataram I., Siegel R.L., Torre L.A., Jemal A. (2018). Global cancer statistics 2018: GOLBOCAN estimates of incidence and mortality worldwide for 36 cancers in 185 countries. CA Cancer J. Clin..

[3] Ryan D.P., Hong T.S., Bardeesy N. (2014). Pacreatic adenocarcinoma. N. Engl. J. Med..

[4] Rahib L., Smith B.D., Aizenberg R., Rosenzweig A.B., Fleshman J.M., Matrisian L.M. (2014). Progecting cancer incidence and deaths to 2030: The unexpected burden of thyroid, liver, and pancreas cancer in the United States. Cancer Res..

[5] Storniolo A.M., Enas N.H., Brown C.A., Voi M., Rothenberg M.L., Schilsky R. (1999). An investigational new drug treatment program for patients with gemcitabine: Results for over 3000 patients with pancreatic canrcinoma. Cancer.

[6] Burris H.A., Moore M.J., Andersen J., Green M.R., Rothenberg M.L., Modiano M.R., Cripps M.C., Portenoy R.K., Storniolo A.M., Tarassoff P. (1997). Improvements in survival and clinical benefit with gemcitabine as first-line therapy for patients with advanced pancreas cancer: A randomized trial. J. Clin. Oncol..

[7] Vaccaro V., Sperduti I., Milella M. (2011). FOLFIRINOX versus gemcitabine for metastatic pancreatic cancer. N. Engl. J. Med..

[8] Von Hoff D.D., Ervin T., Arena F.P., Chiorean E.G., Infante J., Moore M., Seay T., Tjulandin S.A., Ma W.W., Saleh M.N. (2013). Increased survival in pancreatic cancer with nab-paclitaxel plus gemcitabine. N. Engl. J. Med..

[9] Halima N.B., Saad R.B., Khemakhem B., Fendri I., Abdelkafi S. (2015). Oat (Avena satica L.): Oil and Nutrient Compounds Valorization for Potential Use in Industrial Applications. J. Oleo Sci..

[10] Chen C., Wang L., Wang R., Luo X., Li Y., Li J., Li Y., Chen Z. (2018). Phenolic contents, cellular antioxidant activity and antiproliferative capacity of different varieties of oats. Food Chem..

[11] Welch R.W. (1995). The Oat Crop: Production and Utilization.

[12] Anderson J.W., Bridges S.R. (1993). Hypocholesterolemic effects of oat bran in humans. Am. Assoc. Cereal Chem. Int..

[13] Salminen S., Bouley C., Boutron-Ruault M.C., Cummings J.H., Franck A., Gibson G.R., Isolauri E., Moreau M.C., Roberfroid M., Rowland I. (1998). Functional food science and gastrointestinal physiology and function. Brit. J. Nutr..

[14] Zieliński H., Kozłowska H. (2000). Antioxidant activity and total phenolics in selected cereal grains and their different morphological fractions. J. Agric. Food Chem..

[15] Mir S.M., Sahu B.D., Koneru M., Kuncha M., Jerald M.K., Ravuri H.G., Kanjilal S., Sistla R. (2018). Supplementation of oat (Avena sativa L.) extract abates alcohol-induced acute liver injury in a mouse model. Nutr. Res..

[16] Parzonko A., Makarewicz-Wujec M., Jaszewska E., Harasym J., Kozłowska-Wojciechowska M. (2015). Pro-apoptotic properties of (1,3)(1,4)-β-D-glucan from Avena sativa on human melanoma HTB-140 cells in vitro. Int. J. Biol. Macromol..

[17] Baldin V., Lukas J., Marcote M.J., Pagano M., Draetta G. (1993). Cyclin D1 is a nuclear protein required for cell cycle progression in G1. Genes Dev..

[18] Gartel A.L., Tyner A.L. (2002). The Role of the Cyclin-dependent Kinase Inhibitor p21 in Apoptosis. Mol. Cancer Ther..

[19] Toyoshima H., Hunter T. (1994). P27, a novel inhibitor of G1 cyclin-Cdk protein kinase activity, is related to p21. Cell.

[20] Fernald K., Kurokawa M. (2013). Evading apoptosis in cancer. Trends Cell Biol..

[21] Shirwany N.A., Zou M.H. (2014). AMPK: A cellular metabolic and redox sensor. A minireview. Front. Biosci..

[22] Potapova O., Anisimov S.V., Gorospe M., Dougherty R.H., Gaarde W.A., Boheler K.R., Holbrook N.J. (2002). Targets of c-Jun NH(2)-terminal kinase 2-mediated tumor growth regulation revealed by serial analysis of gene expression. Cancer Res..

[23] Mokhtari R.B., Homayouni T.S., Baluch N., Morgatskaya E., Kumar S., Das B., Yeger H. (2017). Combination therapy in combating cancer. Oncotarget.

[24] Dasika G.K., Lin S.C., Zhao S., Sung P., Tomkinson A., Lee E.Y. (1999). DNA damage-induced cell cycle checkpoints and DNA strand break repair in development and turmorigenesis. Oncogene.

[25] Alimbetov D., Askarova S., Unbayev B., Davis T., Kipling D. (2018). Pharmacological Targeting of Cell Cycle, apoptotic and Cell Adhesion Signaling Pathways Implicated in Chemoresistance of Cancer Cells. Int. J. Mol. Sci..

[26] Scjwartz G.K. (2002). CDK inhibitors: Cell cycle arrest versus apoptosis. Cell Cycle.

[27] Henson P.M., Hume D.A. (2006). Apoptotic cell removal in development and tissue homeostasis. Trends Immunol..

[28] Ocker M., Höpfner M. (2012). Apoptosis-modulating drugs for improved cancer therapy. Surg. Res..

[29] Kirsch D.G., Doseff A., Chau B.N., Lim D.S., de Souza-Pinto N.C., Hansford R., Kastan M.B., Lazebnik Y.A., Hardwick J.M. (1999). Caspase-3 dependent cleavage of Bcl-2 promotes release of cytochrome c. J. Biol. Chem..

[30] Edlich F. (2018). BCL-2 proteins and apoptosis: Recent insights and unknowns. Biophys. Res. Commun..

[31] Zhivotovsky B., Burgess D.H., Vanags D.M., Orrenius S. (1997). Involvement of cellular proteolytic machinery in apoptosis. Biochem. Biophys. Res. Commun..

[32] Durkacz B.W., Omidiji O., Gray D.A., Shall S. (1980). (ADP-ribose) n participates in DNA excision repair. Nature.

[33] Aye Y., Li M., Long M.J., Weiss R.S. (2015). Ribonucleotide reductase and cancer: Biological mechanisms and targeted therapies. Oncogene.

[34] Chen Y., Qian X., Liu B. (2011). Advances of drug resistance marker of gemcitabine for non-small cell lung cancer. Zhongguo Fei Ai Za Zhi.

[35] Kurata N., Fujita H., Ohuchida K., Mizumoto K., Mahawithitwong P., Sakai H., Onimaru M., Manabe T., Ohtsuka T., Tanaka M. (2011). Predicting the chemosensitivity of pancreatic cancer cells by quantifying the expression levels of genes associated with the metabolism of gemcitabine and 5-fluorouracil. Int. J. Oncol..

[36] Zhao L.P., Xue C., Zhang J.W., Hu Z.H., Zhao Y.Y., Zhang J., Huang Y., Zhao H.Y., Zhang L. (2012). Expression of RRM1 and its association with resistancy to gemcitabine-based chemotherapy in advanced nasopharyngeal carcinoma. Chin. J. Cancer.

[37] Boukovinas I., Papadaki C., Mendez P., Taron M., Mavroudis D., Koutsopoulos A., Sanchez-Ronco M., Sanchez J.J., Trypaki M., Staphopoulos E. (2008). Tumor BRCA1, RRM1 and RRM2 mRNA expression levels and clinical response to first-line gemcitabine plus docetaxel in non-small-cell lung cancer patients. PLoS ONE.

[38] Yang J., Wang P., Wu W., Zhao Y., Idehen E., Sang S. (2016). Steroidal Saponins in Oat Bran. J. Agric. Food Chem..

[39] Mandeau A., Aries M.F., Boé J.F., Brenk M., Crebassa-Trigueros V., Vaissière C., Teysseyre V., Bieber T. (2011). Rhealba® oat plantlet extract: Evidence of protein-free content and assessment of regulatory activity on immune inflammatory mediators. Planta Med..

[40] Chou T.C., Talalay P. (1984). Quantitative analysis of dose-effect relationships: The combined effects of multiple drugs or enzyme inhibitors. Adv. Enzyme Regul..

